# Application basis of combining antiangiogenic therapy with radiotherapy and immunotherapy in cancer treatment

**DOI:** 10.3389/fonc.2022.978608

**Published:** 2022-11-09

**Authors:** Meng Yuan, Yirui Zhai, Zhouguang Hui

**Affiliations:** ^1^ Department of Radiation Oncology, National Cancer Center/National Clinical Research Center for Cancer/Cancer Hospital, Chinese Academy of Medical Sciences and Peking Union Medical College, Beijing, China; ^2^ Department of VIP Medical Services, National Cancer Center/National Clinical Research Center for Cancer/Cancer Hospital, Chinese Academy of Medical Sciences and Peking Union Medical College, Beijing, China

**Keywords:** cancer, radiotherapy, immunotherapy, antiangiogenic therapy, tumor microenvironment

## Abstract

How to further optimize the combination of radiotherapy and immunotherapy is among the current hot topics in cancer treatment. In addition to adopting the preferred dose-fractionation of radiotherapy or the regimen of immunotherapy, it is also very promising to add antiangiogenic therapy to this combination. We expound the application basis of cancer radiotherapy combined with immunotherapy and antiangiogenic therapy.

## Introduction

Radiotherapy combined with immunotherapy is a highly promising strategy in the field of cancer treatment. However, this combination strategy can also encounter treatment resistance. In addition, there is an unavoidable risk of toxicity overlap with combination therapy. How to optimize the combination therapy regimens is an ongoing focus area of cancer research. Possible approaches include further improving treatment response rates, expanding the potential population who can benefit from treatment, and reducing treatment-related adverse effects. Antiangiogenic therapy can control tumor growth by inhibiting vascular smooth muscle cell differentiation, blocking the formation of abnormal vascular networks, and resisting the proliferation and migration of endothelial cells. In combination treatment, antiangiogenics further enhance treatment efficacy and prolong patient survival (1). A class of antiangiogenic treatment represented by vascular endothelial growth factor (VEGF) inhibitors or associated targeted therapy has been reported to increase radiotherapy sensitivity by downregulating angiogenic factors, normalizing blood vessels, alleviating hypoxia, and adjusting the cell cycle. Antiangiogenic treatment can also balance the tumor microenvironment and eliminate immunosuppressive factors (2–4). When combined with immunotherapy, antiangiogenics can further promote the efficient immune response of activated T cells (5–8). In this article, we review the application basis and progress of cancer radiotherapy combined with immunotherapy and antiangiogenic therapy.

## Angiogenesis in the tumor microenvironment

Angiogenesis plays an important role in the occurrence, progression, and metastasis of cancer. During tumor progression, there is an imbalance in the levels of pro-angiogenic and anti-angiogenic factors. An “angiogenesis switch” is almost always activated and remains on, causing normally quiescent vasculature to continually sprout new vessels. These new vessels are vital for neoplastic growths (9). The characteristics of tumor neovascularization include premature capillary sprouting, curling and excessive branching, distorted and enlarged lumen, unstable blood flow, micro-bleeding, leakage, and abnormal levels of endothelial cell proliferation and apoptosis, resulting in poor perfusion of tumor microenvironment, which further aggravates microenvironment hypoxia, extracellular acidosis, nutrient deficiency, elevates lactate level and adenosine concentrations. The unique metabolic characteristics of the tumor microenvironment are thus formed (9, 10). These metabolic characteristics, dominated by impaired perfusion and hypoxia, are the main driving factors of intra-tumoral heterogeneity, genetic instability, and malignant progression, and can hinder the tumor killing effect of immune cells; on the other hand, abnormal tumor perfusion also compromises the delivery of antitumor drugs and the efficacy of radiotherapy (11).

The known hypoxia regulators of tumor microenvironment include hypoxia inducible factor (HIF), VEGF, platelet-derived growth factor β (PDGF-β), carbonic anhydrase 9 (CA9), erythropoietin (EPO) and p53. Among them, HIF is the most prominent one. HIF is a transcription factor, including HIF-1, HIF-2 and HIF-3, which exists in all human cell types (12, 13). To date, most studies have focused on HIF-1, which is produced by cells under hypoxic stress, and is composed of two heterodimer subunits: HIF-1α and HIF-1β. The expression of HIF-1 is closely related to cancer prognosis (11).

Regarding the mechanism of tumor angiogenesis, VEGF is a critical regulator of the balance between vascular regression and growth (14). Hypoxia and HIF-1α also play an important role in tumor angiogenesis (15, 16). Previous studies have found that HIF-1α is involved in the angiogenesis of non-small cell lung cancer, and the expression levels of HIF-1 and VEGF in lung cancer were significantly higher than those in the adjacent normal tissues. Besides, HIF-1α is positively correlated with the expression of VEGF (17).

To elucidate the abnormal angiogenesis patterns in the tumor microenvironment, Holash and colleagues (14) put forward the “vascular crisis model” hypothesis. This model proposes early overexpression of angiopoietin 2 (ANGPT 2) in the absence of VEGF, which leads to vascular degeneration and microenvironment hypoxia. Once hypoxia occurs, VEGF is upregulated to promote the initiation of angiogenesis. There is another hypothesis proposed by Cao et al. called the “acceleration model” (18). In this model, hypoxia is not responsible for initiating angiogenesis; on the contrary, it is driven by non-hypoxia-mediated mechanisms, such as the up-regulation of VEGF by oncogenes. Once angiogenesis is initiated, tumor cells accelerate proliferation, create hypoxic environment, and HIF-1 is up regulated to further accelerate angiogenesis. Both models comprise the concept of cooption before angiogenesis, and emphasize the importance of hypoxia in uncontrolled tumor angiogenesis. These models also converge on an unchecked angiogenesis cycle, which keyed by perfusion instability, reactive oxygen species (ROS), hypoxia and persistent HIF-1 activity.

## Antiangiogenic therapy sensitizes radiotherapy

Emerging evidence suggests that radiation resistance mediated by hypoxic cells is the main cause of tumor recurrence and metastasis (19). On the one hand, hypoxia impairs the efficacy of radiotherapy by weakening the direct effect of DNA damage. On the other hand, the significant suppressive immune microenvironment of hypoxia affects the efficacy of radiotherapy by indirect and non-targeted ways. Preclinical studies have demonstrated that HIF-1α can independently promote radiation resistance (20); while some other preclinical evidence indicates that VEGF being reactively up regulated in tumors from the beginning of radiotherapy may also be a contributor to the development of radioresistance (21). One probable mechanism for radioresistance is the activation of the PI3K/Akt/mTOR pathway which in turn helps activate the HIF-1α-VEGF pathway in irradiated tumor cells (21, 22). Therefore, alleviating hypoxia and balancing tumor microenvironment are keys to reduce radiotherapy resistance.

Vascular normalization is an essential means to alleviate hypoxia in the tumor microenvironment. Once the imbalance between the anti-angiogenic and pro-angiogenic factors in the tumor microenvironment return to baseline, vascular normalization can occur. The process of tumor vascular normalization involves an increase in tumor vascular cell tight connection and vascular pericyte coverage, as well as enhanced vascular integrity, which can remedy the disorganized tumor vasculature and therefore improve the blood circulation in tumor tissue. Preclinical and clinical evidence suggests that the normalization of abnormal tumor vasculature can reduce tumor hypoxia, tissue hyperpermeability, and interstitial fluid pressure. Vascular normalization facilitates not only the delivery of exogenous agents, but also the efficacy of radiotherapy, and the immunity of immune cells (23). The increase of oxygen content in hypoxic tumor tissue can increase reactive oxygen species after irradiation, resulting in increased DNA damage, abnormal mitosis, and ultimately cell death (4). In addition, the reduction in microenvironment hypoxia can further increase the efficacy of radiotherapy by balancing immunosuppressive factors, regulating energy metabolism, apoptosis, and cell proliferation (11, 24).

## Combining radiotherapy and immunotherapy

The rationale for combining radiotherapy and immunotherapy is comprehensive. On the one hand, immune checkpoint inhibitors (ICI) can enhance the anti-tumor immunity caused by radiotherapy. Radiotherapy alone cannot always produce a sufficient anti-tumor immune response due to the immunosuppressive microenvironment already existing in the tumor. Therefore, the abscopal effect rarely occurs clinically when radiotherapy is administered alone. ICIs including CTLA-4 and PD-1/PD-L1 monoclonal antibodies can enhance the radiation-induced *in situ* vaccination. The combination of radiotherapy and immunotherapy has obvious synergistic effects and may shift the tumor immune system balance toward elimination. Combination therapy can thus effectively suppress tumor growth and metastasis, which is better than either radiotherapy or ICI alone (25, 26).

Radiotherapy also effectively increases the effect of ICIs. Besides substantially reducing tumor burden, radiotherapy can potentiate the local and systematic effect of immunotherapy. Although CTLA-4 and PD-1/PD-L monoclonal antibodies can reverse the inhibitory effect of T cells to some degree in the immunosuppressive tumor microenvironment, the activation of effector T cells still depends on antigen exposure, stimulation, and participation of activated costimulatory molecules on the surface of mature antigen presenting cells (APC). Radiotherapy can enhance tumor antigen expression and presentation, and hence extend the immune response induced by ICIs. In addition, radiotherapy can potentiate the immunomodulatory effect by increasing cytotoxic T lymphocyte (CTL) infiltration, enhancing CTL activity, enlarging the antigen peptide pool, and triggerring the diffusion of tumor antigen determinants (27). Deng et al. (25) found that radiotherapy combined with anti-PD-L1 monoclonal antibody can reduce myeloid-derived suppressor cells (MDSC) and their immunosuppressive effect. Radiotherapy can upregulate PD-L1 expression in tumor cells and PD-1 in T cells, which is also a significant mechanism for the combination with PD-1/PD-L1 monoclonal antibody (28).

## Mechanisms of combining antiangiogenic therapy with immunotherapy

Angiogenesis inhibitors can cause the degeneration of existing tumor blood vessels, prevent the formation of abnormal new blood vessels, and normalize the tumor vascular system (29, 30). The recovery of blood flow and oxygenation can not only improve drug delivery, but also bring a higher level of glucose, thus increasing the availability of amino acids (31, 32). Tumor-infiltrating T cells need sufficient glucose, oxygen, and amino acids to maintain optimal activity; therefore, the combination of immunotherapy and antiangiogenetic therapy in the clinic is reasonable. On the other hand, the main steps involved in the tumor immune response include tumor antigen release, APCs capturing, processing antigens and activating T cells, the effector T cells interacting with vascular endothelial cells, and finally migrating across the endothelium into tumor areas to produce an antitumor response.

Antiangiogenetic therapy can enhance the tumor immune response by promoting the maturation and activity of dendritic cells and the infiltration of T cells. VEGF-A inhibits dendritic cell differentiation and activation, and suppresses effector T cell responses (33, 34). In patients with colorectal cancer, lung cancer, or breast cancer treated with bevacizumab, the number of immature dendritic cells decreased, and the antigen-presenting effect of dendritic cells increased (35). Increased VEGF levels can lead to “incompetence” of endothelial cells, which is characterized by the downregulation of adhesion molecules such as ICAM-1, thus reducing the adhesion and infiltration of T cells and other immune cells into the tumor (36, 37). The use of antiangiogenic agents can reverse endothelial cell incompetence and increase the infiltration of lymphocytes into tumors (38). Preclinical studies have shown that the regulation and normalization of tumor blood vessels increases T cell infiltration (39). Treatment with VEGF inhibitors can increase T cell recruitment and tumor infiltration (40), and have a synergistic role with anti-PD-1 treatment (41).

## The basis of radiotherapy combined with immunotherapy and antiangiogenetic therapy

In recent years, rapid strides have been made in anti-cancer treatment. However, due to the inherent characteristics of cancer, changing only one aspect of tumor biology is unlikely to have a significant impact on total tumor control. In addition, solid tumor cells have extensive heterogeneity, and inhibition of a certain target or aspect cannot cover all malignant cells. One way to address this dilemma is to combine different treatment strategies that inhibit or affect tumor growth, such as combining radiotherapy, immunotherapy, and antiangiogenetic therapy.

The direct effect of radiotherapy is to cause tumor DNA damage and reduce cell infiltration by inhibiting proliferation and mediating cell death. On the other hand, radiotherapy can produce indirect and non-targeted effects, which can promote anti-tumor immune response by destroying cancer cells, enhancing antigen expression, and promoting *in situ* vaccination. Nevertheless, this effect is often offset by the highly immunosuppressive tumor microenvironment. Immunotherapy can restore the host’s anti-tumor immunity to a certain extent, and have a synergistic effect together with radiotherapy. In addition, radiotherapy can up regulate the expression of PD-L1 in tumor cells and PD-1 in T cells. PD-1 pathway induces radiation resistance and limits the efficacy of radiotherapy to a certain degree by inhibiting immune response. PD-1/PD-L1 inhibitors can reverse this state and reduce radiation resistance. However, there are still some challenges in this combination modality of the two treatments, such as the aggravation of tissue hypoxia during hypofractionated radiotherapy, which is often considered a preferred scheme when combined with immunotherapy. A large number of abnormal blood vessels seriously affect tissue perfusion, drug delivery, lymphocyte infiltration, counteract the positive factors in the tumor microenvironment and further aggravate immunosuppression.

**Figure 1:**
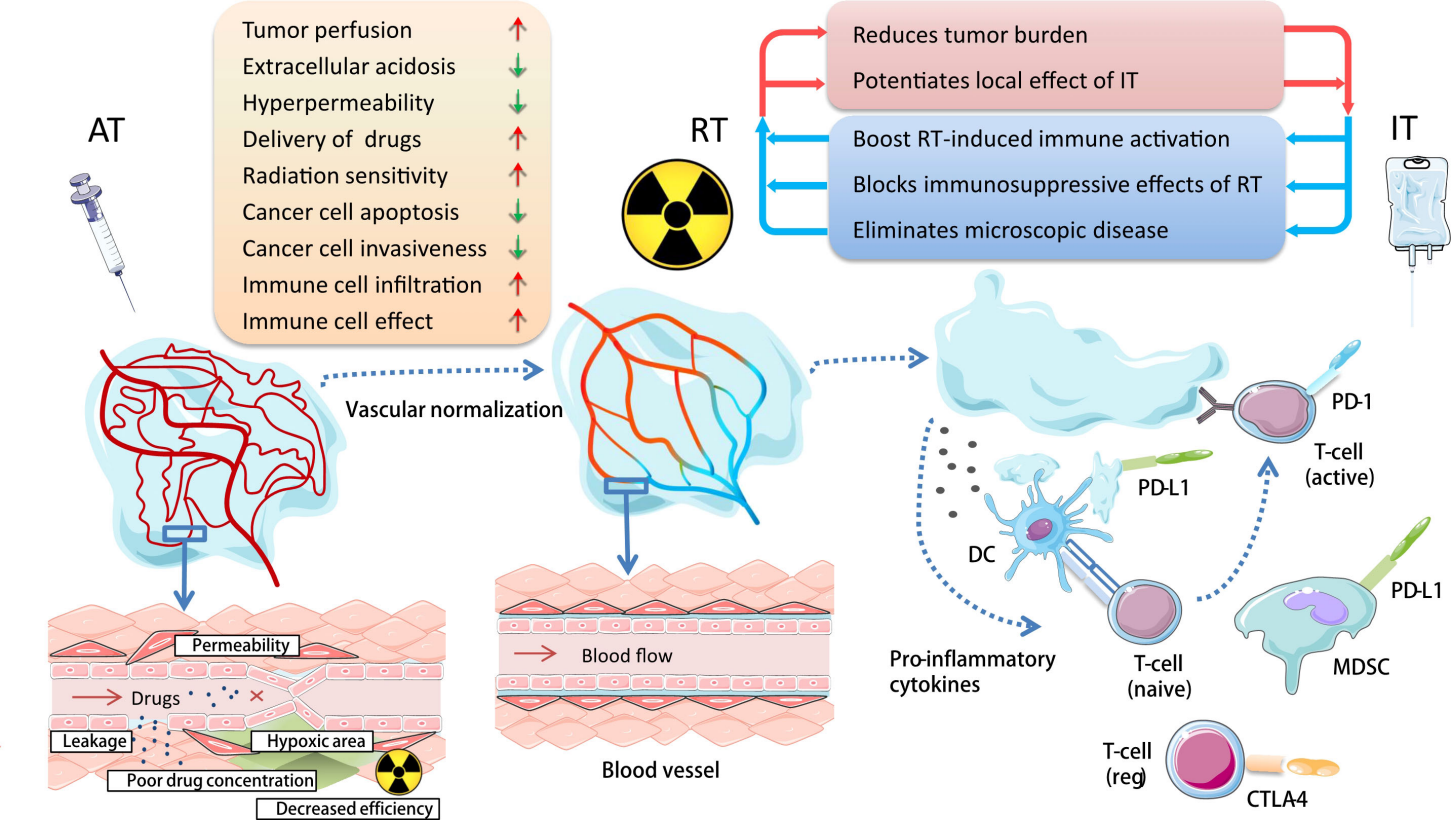
Basis for combining antiangiogenic therapy with radiotherapy and immunotherapy in cancer treatment. Antiangiogenic therapy can reconstruct and regulate the tumor vascular system; reduce leakage, tumor hypoxia, and tissue interstitial fluid pressure; and improve drug delivery and balance tumor immune microenvironment, so as to make the tumor more sensitive to radioimmunotherapy. AT, antiangiogenic therapy; RT, radiotherapy; IT, immunotherapy; DC, dendritic cell; MDSC, myeloid-derived suppressor cell.

Antiangiogenic therapy can balance angiogenesis in the tumor microenvironment, reduce abnormal angiogenesis, reconstruct and regulate the tumor vascular system, reduce leakage, tumor hypoxia and tissue interstitial fluid pressure, and improve drug delivery, to make the tumor more sensitive to radiotherapy, chemotherapy and immunotherapy (42, 43). Additionally, some studies have reported that angiogenesis inhibitors downregulate TGF-β1 induced by radiotherapy and other inflammatory molecules, and therefore, reduce the degree of radiation-induced lung injury to a certain extent (44), which can make more patients benefit from the combination of radiotherapy and immunotherapy. The combination of the three treatments has rich theoretical basis (**Figure 1**), and is expected to bring a brilliant future to the cancer treatment.

**Figure 2:**
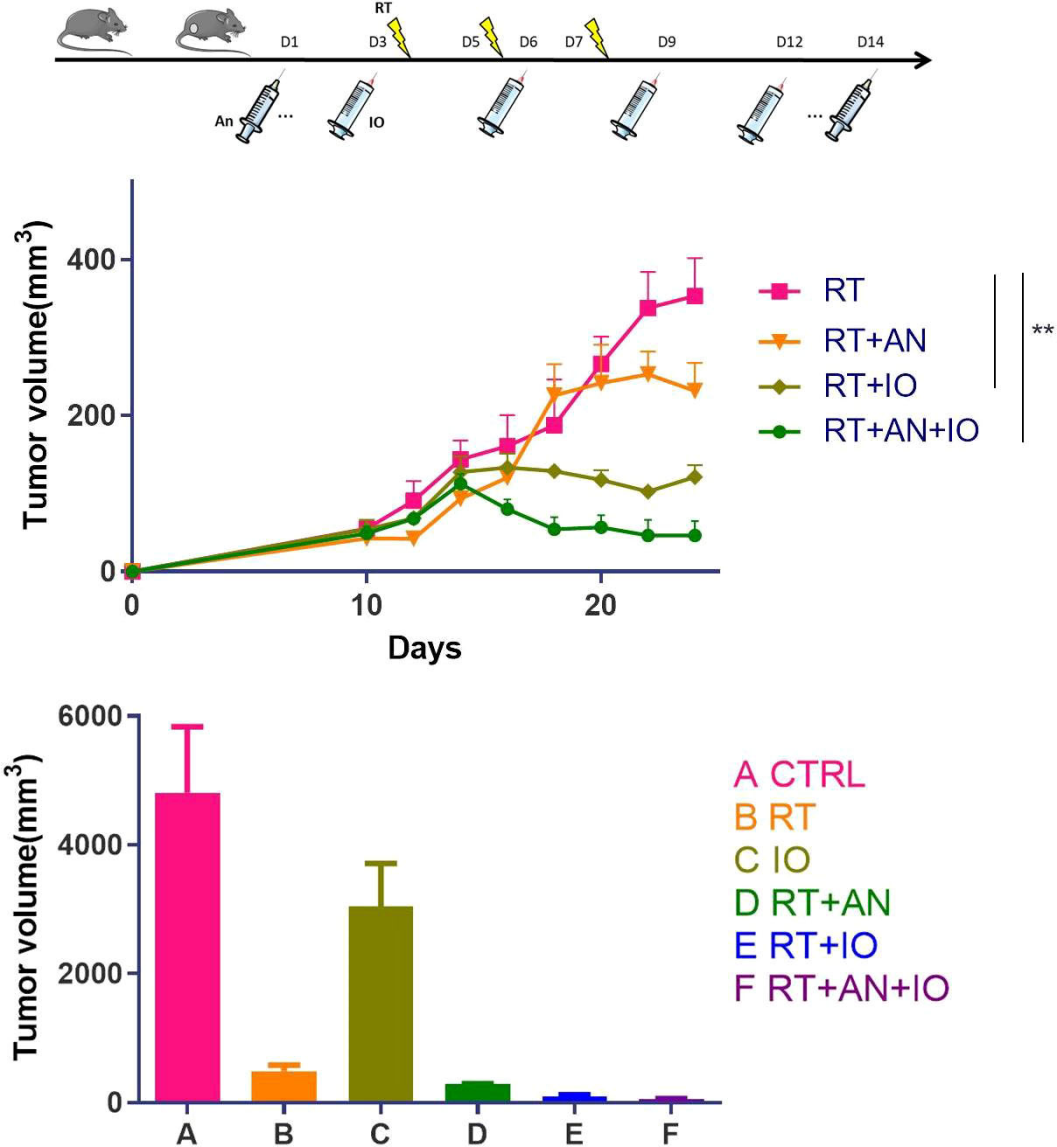
Radiotherapy combined with anti-PD-L1 and anlotinib showed the most remarkable antitumor effect in murine lung cancer. CTRL, control; RT, radiotherapy; IO, immunotherapy; AN, anlotinib. Adapted from our published article (ref. 45), copyright 2022 Meng Yuan et al., with permission from Hindawi. ^∗∗^ represents p values < 0.01.

In our recent study (45), to explore the role of anlotinib (AL3818, an antiangiogenic targeted drug) in the combination of radiotherapy and immune checkpoint inhibitors, we established the C57BL/6 mouse subcutaneous tumor model. To better replicate the clinical scenario, we treated Lewis lung cancer (LLC)-bearing mice with various regimens: radiotherapy (RT), anti-PD-L1, RT+anlotinib, RT+anti-PD-L1, or RT+anti-PD-L1+anlotinib. In previous studies, hypofractionated radiotherapy exhibited more dramatic synergetic effect combined with immunotherapy than single high dose irradiation (46), and therefore, the irradiation was administered as three fractions of 8 Gy. The first agent of anlotinib was given 2 days before RT, considering of priming with the antiangiogenic therapy to produce a “treatment window”, and was given daily for up to 14 days. We performed anti-PD-L1 antibody injections concurrently with and following hypofractionated radiotherapy to make a better synergy. We observed that both radioimmunotherapy and the triple therapy significantly inhibited tumor growths compared with radiation alone. Triple therapy exhibited better antitumor efficacy than radioimmunotherapy and made the best antitumor strategy (**Figure 2**).

Furthermore, using flow cytometry and multiplex immunofluorescence, we found that the addition of anlotinib boosted the infiltration of CD8+ T cells and M1 cells, while causing a decrease in MDSCs and M2 cells compared with radioimmunotherapy. Multiplex immunoassay have showed that the levels of IFN-γand IL-18, which were associated with positive immune responses, were the highest in the triple therapy group. IL-1 beta, IL-2, IL-6, IL-10, IL-13, IL-23 and Arg-1, most of which were related to suppressive tumor immune microenvironment, were significantly reduced. The trinity treatment increased tumor-infiltrating lymphocytes and further reversed the immunosuppressive effect in the tumor microenvironment. In addition, the common regulators of tumor immune microenvironment NF-κB, MAPK, and AKT pathways were downregulated in triple therapy compared with radioimmunotherapy. Based on these preliminary translational observations, this work demonstrated that antiangiogentic therapy could produce a synergistic effect when combined with radioimmunotherapy.

Two other studies have demonstrated the superior efficacy of combining antiangiogenic therapy with radiotherapy and immunotherapy. In a study by Pierini (42), mice bearing subcutaneous CT26 colorectal or TC1 lung tumors were treated with heterologous TEM1 (a protein expressed in the tumor-associated endothelium and/or stroma of various types of cancer) -based vaccine, in combination with RT, and PD-L1 antibody or combinations of these therapies. Heterologous TEM1 vaccine and RT combination therapy boosted tumor-associated antigen cross-priming and augmented PD-1/PD-L1 signaling. Blocking the PD-1/PD- L1 axis in combination with dual therapy further increased the antitumor effect. Additionally, Chen and colleagues (43) found that more prominent effects were observed when anti-VEGF was added to RT + anti-PD-L1 therapies. Phenotypic analyses revealed that anti-cancer treatments increased the proportion of effector memory T cells in tumor-infiltrating lymphocytes and splenocytes, and RT in combination with anti-VEGF and anti-PD-L1 therapies, further increased the proportion of central memory T cells in splenocytes.

## Clinical status of the trinity strategy and future directions

Recently, several clinical trials have just initiated to evaluate the efficacy and safety of the combination of antiangiogenic therapy, immunotherapy, and radiotherapy (**Table 1**). This combination strategy covers locally advanced, advanced, and recurrent cancer. Associated cancer types include lung cancer, gastric/gastroesophageal junction cancer, nasopharyngeal cancer, gliomas and rectal cancer. All these trials are phase 1/2. Several different dose fractionation regimens of radiotherapy are adopted. Anti-PD-1/PD-L1 monoclonal antibody is the most used immunotherapy agent. Antiangiogenic drugs in the clinical trials include bevacizumab, anlotinib and endostatin, with the latter two used concurrent with radiotherapy. From these clinical studies, this triple combination model is still in the exploratory stage. The optimal dose fractionation regimen of radiotherapy is unknown, and it is also worth exploring whether it is possible to reduce the field of irradiation and lower the dose without compromising efficacy in this treatment combination. In addition, the combination of multiple treatment modalities is likely to increase adverse events and therefore, safety should be a key concern when selecting agents of immunotherapy and antiangiogenic therapy (47).

**Table 1:** Clinical trials of combining antiangiogenic therapy with radiotherapy and immunotherapy.

Trial ID	Title	Start date	phase	Treatment	Radiotherapy	Immunotherapy	Antiangiogenic therapy	Primary endpoint
NCT05468242	Study of Tislelizumab for Locally Advanced or Oligometastatic Non-Small Cell Lung Cancer Following Neoadjuvant Chemotherapy Plus Tislelizumab ± Bevacizumab and Definitive Concurrent Chemoradiation Therapy ± Anlotinib	February 1, 2022	2	RT + IT + AT + CT	Hypofractionated radiation	Tislelizumab	Bevacizumab, Anlotinib	ORR, PFS
NCT05387681	Preoperative Short Course Radiotherapy With Envafolimab, Endostatin and SOX Regimen in Locally Advanced Gastric/Gastroesophageal Junction Adenocarcinoma	May 30, 2022	2	RT + IT + AT + CT	25Gy/5Gy/5F	Envafolimab	Endostatin	pCR
NCT05341193	PD-1 Blockade and Bevacizumab Replace Cisplatin in Locoregionally Advanced Nasopharyngeal Carcinoma	April 30, 2022	1/2	RT + IT + AT + CT	Conventional fractionated radiotherapy	Toripalimab	Bevacizumab	Safety
NCT03532295	Retifanlimab and Epacadostat in Combination With Radiation and Bevacizumab in Patients With Recurrent Gliomas	April 20, 2020	2	RT + IT + AT +/- epacadostat (IDO inhibitor)	35Gy/3.5Gy/10F	Retifanlimab	Bevacizumab	OS
NCT04017455	Neoadjuvant Treatment in Rectal Cancer With Radiotherapy Followed by Atezolizumab and Bevacizumab (TARZAN)	October 22, 2019	2	RT + IT + AT	Dose not mentioned	Atezolizumab	Bevacizumab	cCR
NCT02829931	Hypofractionated Stereotactic Irradiation With Nivolumab, Ipilimumab and Bevacizumab in Patients With Recurrent High Grade Gliomas	August 22, 2016	1	RT + IT + AT	Hypofractionated Stereotactic Irradiation	Ipilimumab + Nivolumab	Bevacizumab	Safety and tolerability

RT, radiotherapy; IT, immunotherapy; AT, antiangiogenic therapy; CT, chemotherapy.

## Author contributions

ZH: conceptualization. MY: original draft. ZH and YZ: review. All authors contributed to the article and approved the submitted version.

## Funding

CAMS Innovation Fund for Medical Sciences (CIFMS: 2020-I2M-C&T-B-074); Capital’s Funds for Health Improvement and Research (2022-1-4022); Beijing Hope Run Special Fund of Cancer Foundation of China (ZZ2021A02); Beijing Xisike Clinical Oncology Research Foundation (Y-HR2020ZD-0779).

## Conflict of interest

The authors declare that the research was conducted in the absence of any commercial or financial relationships that could be construed as a potential conflict of interest.

## Publisher’s note

All claims expressed in this article are solely those of the authors and do not necessarily represent those of their affiliated organizations, or those of the publisher, the editors and the reviewers. Any product that may be evaluated in this article, or claim that may be made by its manufacturer, is not guaranteed or endorsed by the publisher.
